# Hybrid Piezo/Magnetic Electromechanical Transformer

**DOI:** 10.3390/mi12101214

**Published:** 2021-10-05

**Authors:** Adrian A. Rendon-Hernandez, Spencer E. Smith, Miah A. Halim, David P. Arnold

**Affiliations:** Interdisciplinary Microsystems Group (IMG), University of Florida, Gainesville, FL 32611, USA; arendonhernandez@ufl.edu (A.A.R.-H.); smithspencer12@ufl.edu (S.E.S.); md.miah@ufl.edu (M.A.H.)

**Keywords:** electromechanical transformer, electrodynamic transduction, piezoelectric transduction

## Abstract

This paper presents a hybrid electromechanical transformer that passively transfers electrical power between galvanically isolated ports by coupling electrodynamic and piezoelectric transducers. The use of these two complementary electromechanical transduction methods along with a high-Q mechanical resonance affords very large transformations of voltage, current, or impedance at particular electrical frequencies. A chip-size prototype is designed, simulated, fabricated, and experimentally characterized. The 7.6 mm × 7.6 mm × 1.65 mm device achieves an open-circuit voltage gain of 31.4 and 48.7 when operating as a step-up transformer at 729.5 Hz and 1015 Hz resonance frequencies, respectively. When operating as a step-down transformer, the resonance frequencies and the corresponding voltage gains are 728 Hz, 1002 Hz, and 0.0097, 0.0128, respectively. In one operational mode, the system shows a minimum power dissipation of only 0.9 µW corresponding to a power conversion efficiency of 11.8%.

## 1. Introduction

Electromechanical devices using either piezoelectric or electrodynamic transduction have been proposed as an alternative to conventional magnetic transformers. These devices make use of a mechanical vibration or motion for passive transformation of voltage, current, or impedance [1,2]. To maximize end-to-end power transfer efficiency, these often rely on a resonance of high mechanical quality factor resonator, such that mechanical energy losses are minimized.

The notion of a piezoelectric transformer (PT) using a single piezoelectric body was first presented in a patent application by C. A. Rosen et al. in 1954 [3]. PTs convert electrical energy from one circuit into electrical energy in another circuit by using acoustic energy (in the form of mechanical vibrations) from certain piezoelectric materials driven at resonance. PTs are widely used in applications requiring small size, high step-up voltages, and good electromagnetic compatibility (EMC) [4,5]. PTs have also been considered for step-down applications such as AC adapters for battery chargers and the switch-mode power supplies of electronic devices [6,7,8].

Recently, there was significant additional interest in using PTs for ultra-low-power wake-up circuits whose applications extend to various domains such as healthcare, smart cities, industrial monitoring, agriculture, and security surveillance [9,10]. Bassirian et al. [11] presented two MEMS-based piezoelectric resonators integrated in a wake-up receiver with 7 nW of power consumption. Yadav et al. [12] reported on a wake-up receiver that uses piezoelectric transduction and dissipates only 4.4 µW.

Few studies have been published on the potential of PTs as key elements for ultra-low voltage start-up circuits for energy harvesting applications [13,14]. Camarda et al. [15] proposed a MEMS-based PT that achieves a maximum voltage gain of 58 mV/V at 36.3 kHz. Martinez et al. [16] presented a start-up converter that uses PT and achieves a voltage gain of 23.2 at 55.8 kHz. PTs are rarely used in low-frequency (<1 kHz) applications. One reason for this is that each PT topology has an optimum vibration mode—typically in the range of 10 kHz to 1 MHz—that will allow optimum energy transfer through the device [17]. Additionally, Rosen-type PTs exhibit high internal electrical impedance, which may exclude them from low-voltage/high-current applications [18]. In those applications, designing PTs to meet the requirement of step-down power conversion implies the use of various vibration modes such as thickness-extensional and radial modes [19].

In contrast to PTs, our group previously demonstrated the feasibility of an electrodynamic transformer (ET) operating at a very low resonance frequency of only 22 Hz [20]. Like a PT, an ET leverages the mechanical domain to exchange electrical power between input and output ports, but uses electrodynamic transduction (interaction between a permanent magnet and coil) on both ports, in contrast to a PT, which uses piezoelectric transduction [20,21,22]. Electrodynamic transducers generally exhibit much lower electrical impedance (correspondingly lower voltages and larger currents) in comparison to piezoelectric transducers. Additionally, ETs have shown a relatively constant resonance frequency irrespective of the load conditions, which overcomes some frequency tuning challenges commonly found with PTs [22].

**Figure 1:**
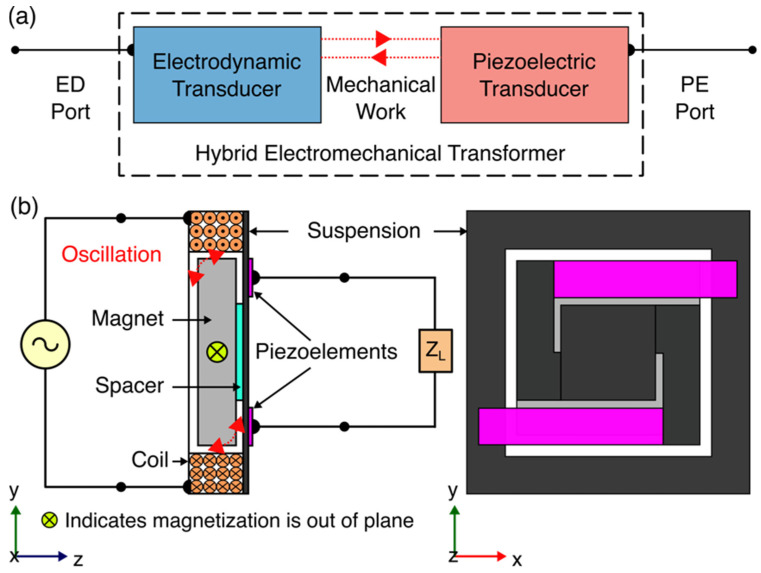
(**a**) Block diagram of a hybrid electromechanical transformer, (**b**) a possible architecture of step-up hybrid electromechanical transformer using electrodynamic and direct piezoelectric transduction on its electrodynamic (ED) and piezoelectric (PE) ports, respectively.

While literature reports vibration energy harvesters using piezoelectric [23], electrodynamic [24], and/or both [25] transduction techniques, here we are focusing on utilizing those transduction techniques, specifically hybridized piezoelectric and electrodynamic transduction, in an electromechanical transformer. Here, we present results on a multi-transduction device, termed “hybrid electromechanical transformer,” which enables sub−1 µW power dissipation, low driving frequency, high voltage gain, and low-profile design. The hybrid electromechanical transformer uses a coupling between magnetic, mechanical, and electrical domains to exchange electrical power between electrodynamic (ED) and piezoelectric (PE) ports, as illustrated in Figure 1a.

## 2. Materials and Methods

This section outlines the prototype implementation of a hybrid electromechanical transformer and its working principles.

### 2.1. Device Overview

Figure 1b presents the architecture for the prototype hybrid electromechanical transformer. The device comprises an oscillating suspension connecting electrodynamic and piezoelectric transducers. A laterally magnetized, square, permanent magnet attached to the suspension through a spacer and surrounded by a square multi-turn coil form the electrodynamic transducer. The piezoelectric transducer comprises two piezoelectric unimorphs electrically connected in series, which are adhered to the clamped arms of the suspension (on the face opposite to the permanent magnet).

In the step-up transformer configuration, an AC voltage source is connected to the ED port while an external load is connected to the PE port. When an electric current circulates in the electromagnetic coil, it induces an electrodynamic force on the permanent magnet. The force provokes the magnet to oscillate, causing a mechanical stress on the suspension and similarly on the piezoelectric elements, which is then converted into electricity by means of the direct piezoelectric effect.

Alternatively, the input signal source and external load may be connected to the PE and ED ports, respectively, to operate this device as a step-down transformer. In step-down transformer mode, when an AC voltage is applied to the PE port, a dynamic mechanical strain is generated by means of the converse piezoelectric effect, and this in turn will stimulate a motion on the magnet causing a flux change in the electromagnetic coil. This flux change induces an electromotive force (emf) in the electromagnetic coil by means of Faraday’s law of induction.

In conventional magnetic transformers, the voltage gain depends on the coil turns ratio. However, in a hybrid electromechanical transformer the voltage gain depends on the magnetic field pattern produced by the magnet, the number of turns in the electromagnetic coil, and the size and shape of both the suspension and the piezoelectric elements. Naturally, this intensifies the design complexity of hybrid electromechanical transformers, and full optimization is beyond the scope of this work. Despite this, the resonance behavior and frequency response of the system are studied by a 3D finite-element analysis (FEA), and then validated by experimental measurements. These results are presented in Section 3. Furthermore, the open-circuit voltage gain for the step-up and step-down type hybrid electromechanical transformer is analyzed in Section 4.

### 2.2. Open-Circuit Voltage Gain and Frequency Response

To determine the open-circuit voltage gain (the ratio of the rms output voltage to the rms input voltage) of the hybrid electromechanical transformer for its two power conversion types, the frequency of the input signal is varied over a certain range including the torsional resonance modes of the suspension. As the oscillating suspension approaches torsional resonance, its torsional amplitude reaches a maximum rotation, leading to peaks in power conversion. In both power conversion types (step-up and step-down transformer), maximum voltage gain and efficiency are expected to be achieved at the torsional resonance of the mechanical suspension and when the external load approaches the output port electrical impedance. In addition, the frequency response of the open-circuit voltage indicates the resonator mechanical quality factor, defined by ${Q=f}_{r}/\left(f_{2}{-f}_{1}\right)$, where *f_r_* is the frequency at resonance (i.e., output voltage is maximum, *V_max_*) and *f_1_*, and *f_2_* are the values of frequency where the output voltage is equal to $V_{\max}\;/\sqrt{2}$, respectively. These results are described in Section 4.

The following considerations should be noted:

- Step-up transformer type: the input current is fixed in an attempt to minimize the input voltage, and thus the input power, at the electromagnetic coil.
- Step-down transformer type: the input power is fixed in order to minimize the input voltage across the piezoelectric elements. Note that the input voltage was adjusted in the vicinity of 1 V while its frequency was varied to maintain a constant supplied power during this test.

### 2.3. Optimum Load Resistance

By connecting a variable load resistance to the output port, the optimum load resistance for maximum power transfer and corresponding efficiency of the transformer can be determined. This analysis will be discussed in Section 4.

## 3. System Simulation

**Figure 2:**
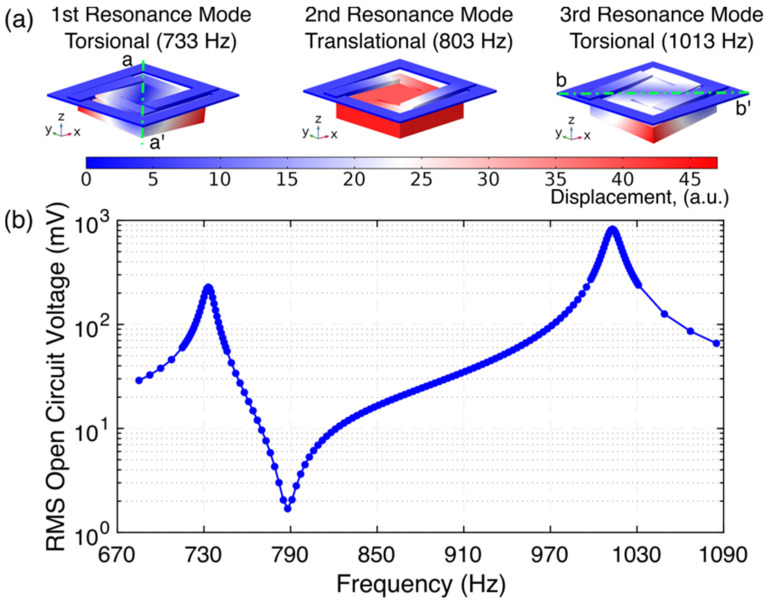
Finite-element analysis (FEA) simulation results for (**a**) the first three mode shapes and associated resonance frequencies of the structure (coil is not shown) and (**b**) the frequency response for the open-circuit output voltage of the hybrid step-up transformer; the applied current is 100 µA_rms_ and the damping ratio *ζ* = 0.0064 (*Q* = 78.1).

A finite-element analysis was carried out using COMSOL Multiphysics^®^ (COMSOL Inc., Stockholm, Sweden). First, a modal study was performed by a 3D model of the system. Figure 2a shows the first three mode shapes and natural frequencies of the system. To simulate the bonding layers (between the magnet, spacer, and mounting platform, and between piezo-elements and suspension arms), a 20 µm-thick elastic layer with Young’s modulus *E* = 2 GPa and Poisson’s ratio v = 0.25 was used. It should be noted that the system exhibited two different torsional modes: natural mode 1 occurred at 733 Hz, which was a torsional rotation about the diagonal axis a-a’, whereas the third mode of vibration at 1013 Hz was a torsional rotation about the diagonal axis b-b’. The second mode shape at 803 Hz corresponded to a displacement mode in the z-direction.

Second, a frequency domain study was carried out to investigate the frequency response of the hybrid electromechanical transformer for its open-circuit output voltage when operated in step-up transformer connection. For this simulation, an AC current signal of 100 µA_rms_ was applied to the electromagnetic coil along with a damping ratio *ζ* = 0.0064, corresponding to a Q-factor of 78.1 (as measured experimentally) applied to the mechanical suspension. It is important to note that an isotropic structural loss factor *η* = 1/Q, was used in COMSOL Multiphysics through the damping subnode under material model. As shown in Figure 2b, the open-circuit voltage, evaluated at the piezoelectric elements, reached two positive peaks: the first one of 228.3 mV_rms_ at 733 Hz, and the second one of 823.4 mV_rms_ at 1013 Hz. A negative peak of 2.3 mV_rms_ was observed near 803 Hz where the system was expected to resonate in vertical displacement along the z-direction.

This study identified two challenges. The first is choosing appropriate dimensions of the piezoelectric elements that would enhance the system performance. More details on a parametric finite element analysis study to explore the output power of the piezoelectric port (i.e., varying the length and thickness of the piezoelectric elements) for a similar device can be found in [26]. The second is designing the suspension and electromagnetic coil for an efficient magnetic flux to reduce power losses. These points are evidence of the need for an optimization design study that maximizes the output voltage gain of the hybrid electromechanical transformer, which will be reserved for future study.

## 4. Experimental Validation

To demonstrate the concept, a hybrid electromechanical transformer prototype was fabricated and experimentally characterized.

### 4.1. Prototype Fabrication

**Figure 3:**
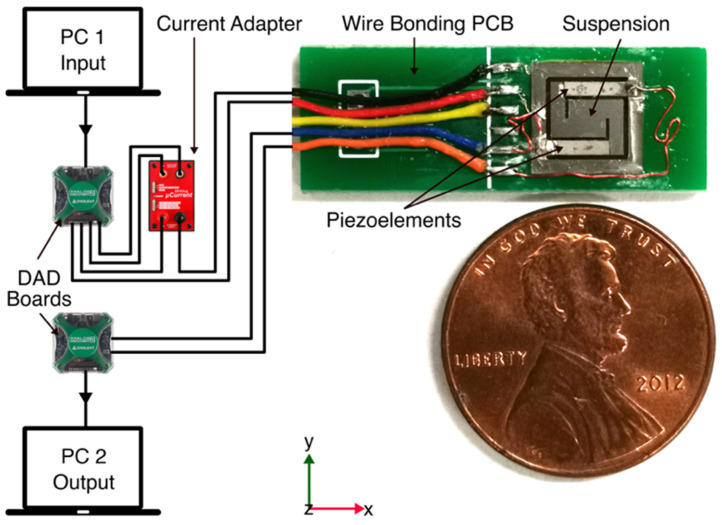
Block diagram and photograph of the experimental testbed for characterizing the hybrid electromechanical transformer with its size compared to a US one-cent coin.

Figure 3 shows the block diagram and photograph of our experimental testbed for testing the hybrid transformer. The suspension structure was fabricated by laser micro-machining 125 µm-thick titanium (Ti) shim stock (McMaster-Carr, Elmhurst, IL, USA). The dimensions of the suspension are 7.6 mm × 7.6 mm. The width of the suspension arms is 1 mm, and the center platform dimensions (where the magnet is attached to via spacer) are 2.6 mm × 2.6 mm. The spacer, made of silicon (Si), was diced out of a 200 µm-thick, double side polished Si wafer (University Wafer, Inc., South Boston, MA, USA). A laterally magnetized permanent magnet NdFeB grade N50 (Super Magnet Man, Pelham, AL, USA) was bonded to one side of the center platform via spacer using cyanoacrylate. On the opposite side, two piezo-ceramic patches, each 5 mm × 1 mm × 127 µm, diced from a PZT-5A4E sheet with sputtered nickel electrodes and poled through their thickness (Piezo.com, Woburn, MA, USA), were bonded to the arms of the suspension using silver epoxy (EO-21M-5, EpoxySet Inc., Woonsocket, RI, USA) to form a series connection between two unimorph piezoceramic transducers. A custom, commercially wound, rectangular, self-supported copper electromagnetic coil (44 AWG, 328 turns, 71 Ω DC resistance) whose inner dimensions are 5.6 mm × 5.6 mm × 1.4 mm was glued using cyanoacrylate to the suspension so that it surrounded the permanent magnet. Finally, the assembled device was bonded using cyanoacrylate into a cutout on a printed circuit board (PCB). The PCB mechanically supported and electrically connected the hybrid electromechanical transformer and its ports, respectively. The electrical connections between PCB terminals and piezoelectric electrodes were made by attaching copper wires using silver epoxy. The soldered connections between these copper wires and corresponding PCB terminals as well as between the coil wires and PCB were used. A USB digital oscilloscope and a waveform generator (Diligent Analog Discovery 2, or “DAD”) were used to supply the input signal and to measure the output response, respectively. A precision current adapter (µCurrent^®^ Gold) was used to measure the microamps level current circulating through the electromagnetic coil.

### 4.2. Step-up Transformer

**Figure 4:**
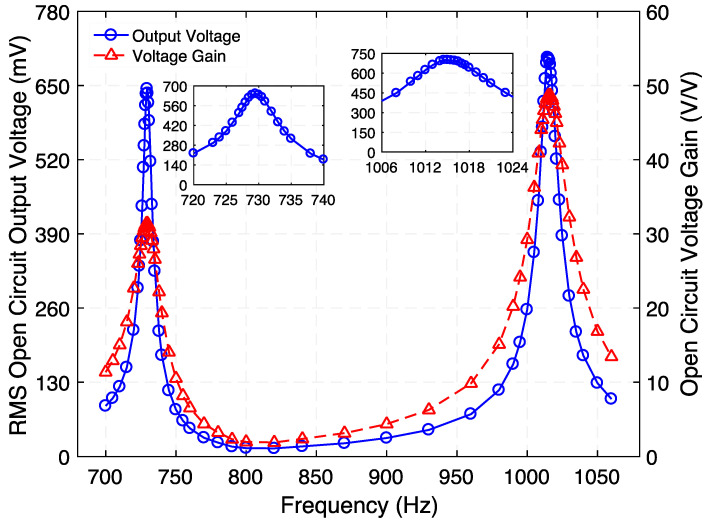
Measured frequency response of the step-up transformer for its rms open-circuit output voltage and open circuit voltage gain. While the peaks at 729.5 Hz and 1015 Hz correspond to the torsional modes of vibration of the structure, the minimum close to 800 Hz corresponds to its translational mode of vibration. The insets show the zoomed-in graph of rms open-circuit output voltage at resonance.

First, when operated as step-up transformer, the frequency response of the open-circuit voltage gain was determined as shown in Figure 4. To do this, an AC voltage signal was generated by an arbitrary waveform generator (Diligent Analog Discovery 2) and was fed into the electromagnetic coil of the transformer’s ED port. The voltage and current were measured using the USB digital oscilloscope built-in on the same data acquisition board. A second DAD board along with a high input impedance (10 MΩ) passive probe were used to measure the output voltage from the transformer’s PE port. This sweep was carried out at a fixed input current (100 µA_rms_). From insets of Figure 4, the output voltage resonance peak indicates a Q value of 112.2 (Mode 1) and 78.1 (Mode 3). Figure 4 shows that two peaks in the open-circuit voltage gain of 31.4 (29.9 dB) and 48.7 (33.7 dB) were achieved at 729.5 Hz and 1015 Hz, respectively. Additionally, a minimum voltage gain of 1.9 (5.5 dB) was observed close to 820 Hz, where the translational resonance of the suspension is expected to occur. The frequencies of the resonance modes closely match with those predicted by the 3D-FEA model (733 Hz, 803 Hz, and 1013 Hz).

**Figure 5:**
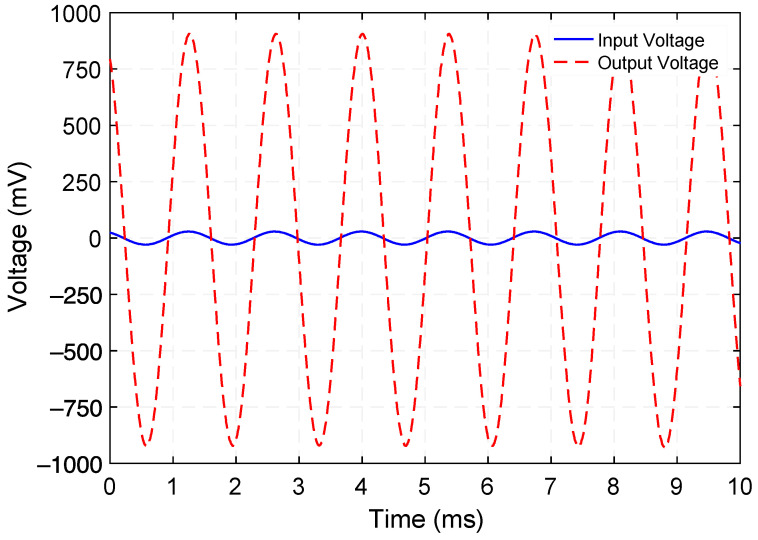
Measured input and open-circuit output voltage waveforms generated by the step-up transformer operating at 729.5 Hz.

**Figure 6:**
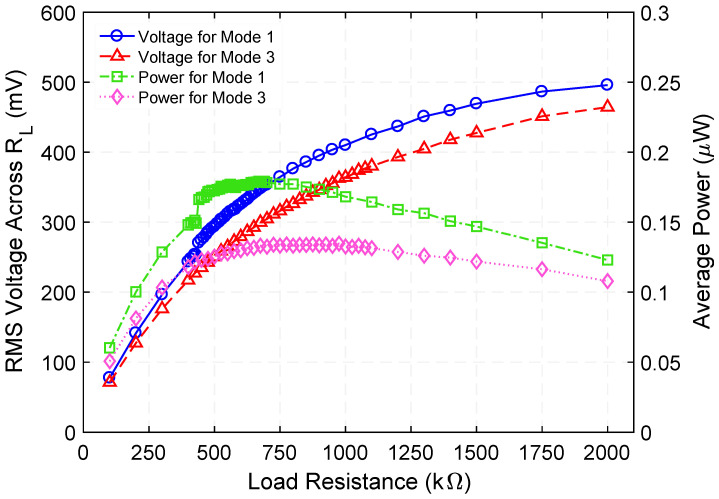
Measured rms voltage across, and time-average power delivered to a variable load resistance when the step-up transformer is operated at 729.5 Hz (Mode 1) and 1015 Hz (Mode 3) resonance frequency.

**Figure 7:**
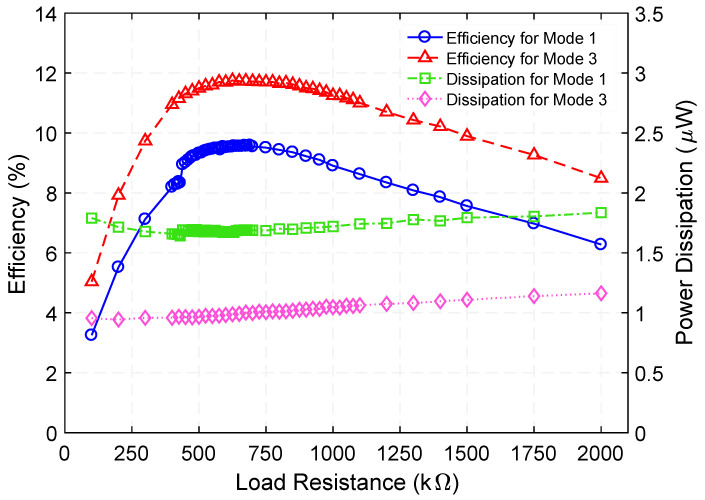
Measured efficiency and power dissipation as a function of load resistance when the step-up transformer is operated at 729.5 Hz (Mode 1) and 1015 Hz (Mode 3) resonance frequency.

Figure 5 presents the measured voltage waveforms generated by the step-up transformer when operating at 729.5 Hz. Figure 6 shows the rms voltage and corresponding time-average power delivered to various load resistances (within the range of 100 kΩ to 2000 kΩ) while a 100 µA_rms_ constant-amplitude alternating input current at frequencies of 729.5 Hz (Mode 1) and 1015 Hz (Mode 3) was maintained. Making use of the measured rms voltage (V_rms_), the time-average power was calculated by using $V_{rms}^{2}/R_{L}$, where *R_L_* is the load resistance value. These experiments reveal that a maximum power of 0.18 µW and 0.13 µW are delivered to an optimum load resistance (for maximum power transfer) of 675 kΩ and 875 kΩ, respectively. Exploiting these findings, the efficiency was estimated by using the ratio of the power delivered to *R_L_* to the power supplied to the system, i.e., $P_{out}/P_{in}$. The input power was obtained by measuring the time-average product of the input current and voltage waveforms. Figure 7 presents the measured efficiency and power dissipation of the step-up transformer. As seen in Figure 7, efficiency peaks and associated power dissipation of 9.6%, 1.7 µW (Mode 1) and 11.8%, 0.9 µW (Mode 3) are reached when load resistance of 690 kΩ and 625 kΩ are used, respectively.

**Figure 8:**
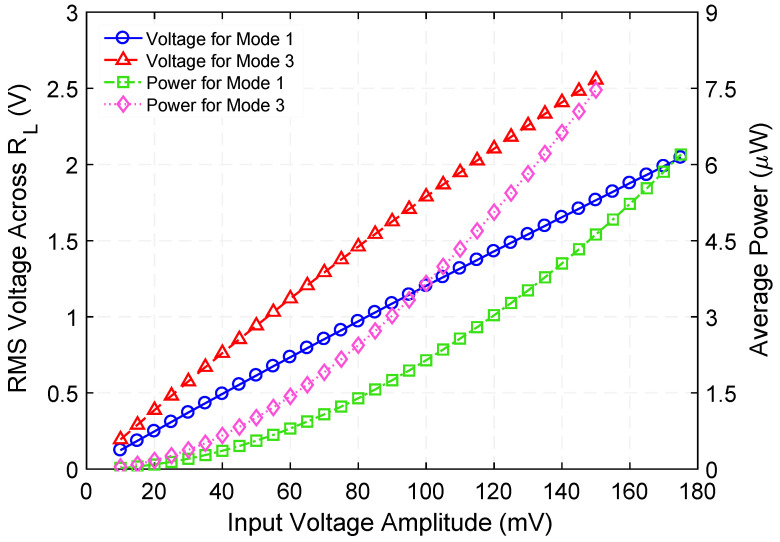
Measured rms voltage across, and time-average power delivered to corresponding optimum load resistance as a function of input voltage when the step-up transformer is operated at 729.5 Hz (Mode 1) and 1015 Hz (Mode 3) resonance frequency.

Next, the voltage across and the time-average power delivered to the optimum load resistances were measured by varying the amplitude of an AC input voltage (within the range of 10 mV to 175 mV) with a constant frequency of 729.5 Hz and 675 kΩ as the load resistance. This experiment was then replicated under conditions in which the input voltage ranged from 10 mV to 150 mV, at 1015 Hz resonance frequency and 875 kΩ load resistance, as shown in Figure 8. As expected, the time-average power shows a quadratic behavior as the input voltage increases.

**Figure 9:**
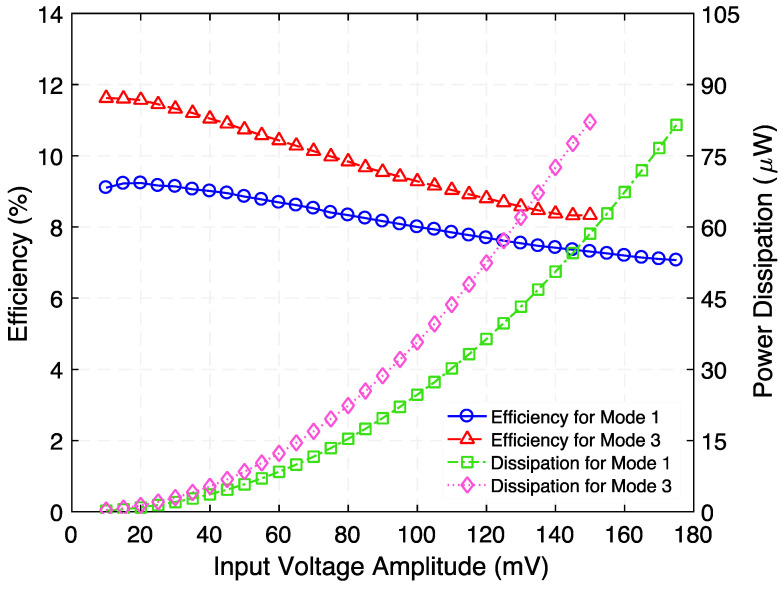
Measured efficiency and power dissipation as a function of input voltage of the step-up transformer when load resistances and resonance frequencies are 675 kΩ and 875 kΩ, and 729.5 Hz (Mode 1) and 1015 Hz (Mode 3), respectively.

Moreover, the efficiency and power dissipation as a function of input voltage for each torsional resonance mode (optimum load connected) were calculated as reported in Figure 9. We believe that the power loss is primarily attributable to the mechanical damping of the suspension and resistance of the electromagnetic coil. In the future, efficiency can be improved by a material optimization for the mechanical resonator —with a very high-quality factor— and optimizing the dimensions and shape of the coil and magnetic circuit.

**Figure 10:**
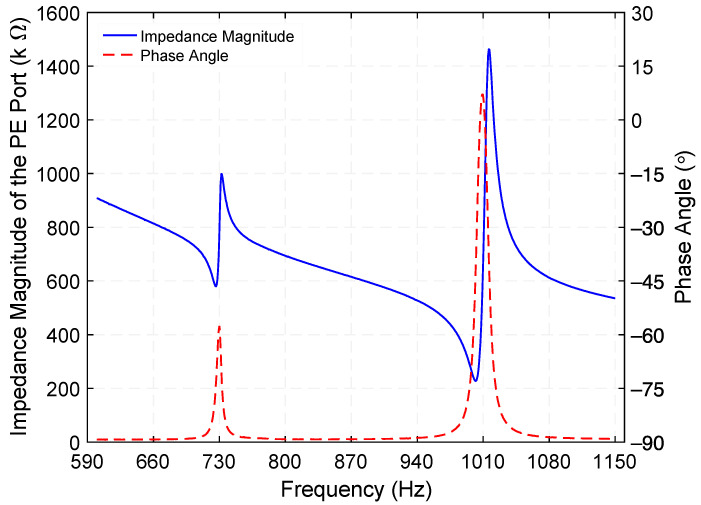
Measured electrical impedance magnitude and phase angle of the piezoelectric (PE) port, an excitation of 500 mV is applied while the electrodynamic (ED) port is open.

Knowledge of the electrical impedance of the two ports is important for end applications. Using a precision impedance analyzer (Hewlett Packard 4294A), the electrical impedance of the PE port was measured. Figure 10 presents the electrical impedance magnitude and phase angle of the PE port when the ED port is open. While two electrical impedance minima of 580.1 kΩ at 726.5 Hz and 227.4 kΩ at 1002 Hz are associated with the torsional resonance frequencies of the PE transducer, two electrical impedance maxima of 998.7 kΩ at 732 Hz and 1.46 MΩ at 1015.9 Hz correspond to its torsional antiresonance frequencies. Substituting these results into $k_{\mathrm{eff}}=\surd \left(f_{a}^{2}{-f}_{r}^{2}\right)/f_{a}$, where *f_r_* and *f_a_* are the resonance and anti-resonance frequencies, respectively, the effective electromechanical coupling coefficient (*k_eff_*) of the PE transducer was determined as 12.2% (Mode 1) and 16.4% (Mode 3).

### 4.3. Step-down Transformer

**Figure 11:**
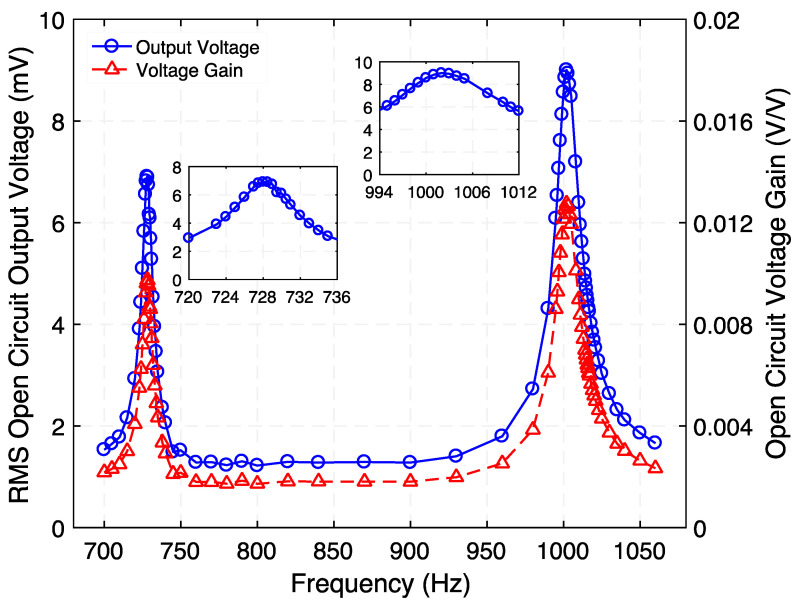
Measured frequency response of the step-down transformer for its rms open-circuit output voltage and open circuit voltage gain. The peaks at 728.5 Hz and 1002 Hz correspond to the torsional modes of vibration of the structure. The insets show the zoomed-in graph of rms open-circuit output voltage at resonance.

**Figure 12:**
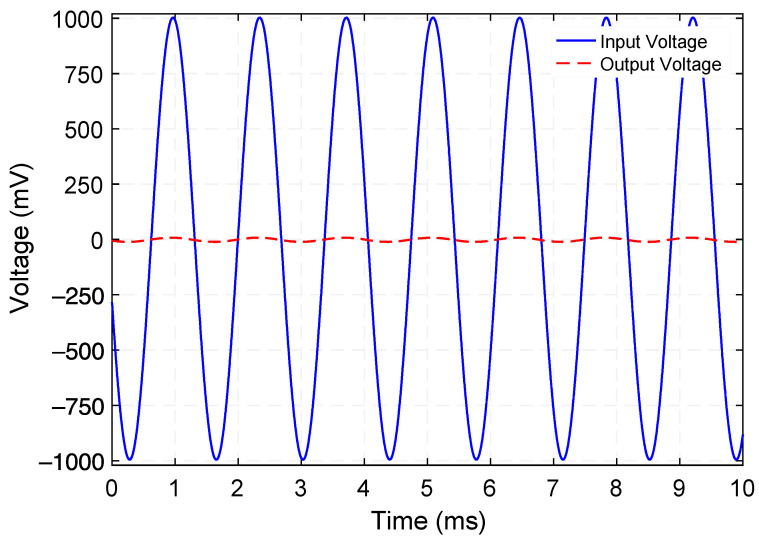
Measured input and open-circuit output voltage waveforms generated by the step-down transformer operating at 728 Hz.

After the characterization of the step-up transformer type was complete, the device was then characterized as a step-down transformer. First, the frequency response of the hybrid electromechanical transformer was examined, as shown in Figure 11. The PE port was used to feed the input voltage signal while the ED port read out the output signals. As stated above, the input power was kept constant (500 µW) to minimize the voltage across the piezoelectric elements. It can be seen from Figure 11 that this response is representative of a second-order system, as expected from the dynamics of the mechanical resonator. It is apparent from insets of Figure 11 that the mechanical quality factor Q is 107.1 and 65.1 for Mode 1 and Mode 3, respectively. Figure 10 shows that two open-circuit voltage gain peaks of 0.0097 (−40.2 dB) and 0.0128 (−37.8 dB) occur at 728 Hz and 1002 Hz, respectively. A possible reason for this shift in resonance frequencies with respect to those reported for the step-up transformer type (729.5 Hz and 1015 Hz) may be in part due to the nonlinear resonance effect—resonance frequencies depend on the amplitude of oscillations. Lastly, Figure 12 shows the measured input and open-circuit output voltage waveforms generated by the step-down transformer operating at 728 Hz.

**Figure 13:**
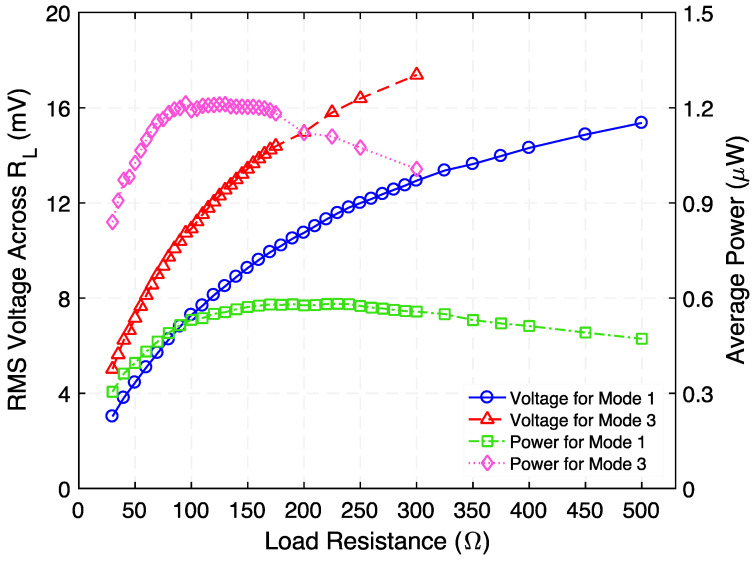
Measured rms voltage across and time-average power delivered to a variable load resistance when the step-down transformer is operated at 728.5 Hz (Mode 1) and 1002 Hz (Mode 3).

**Figure 14:**
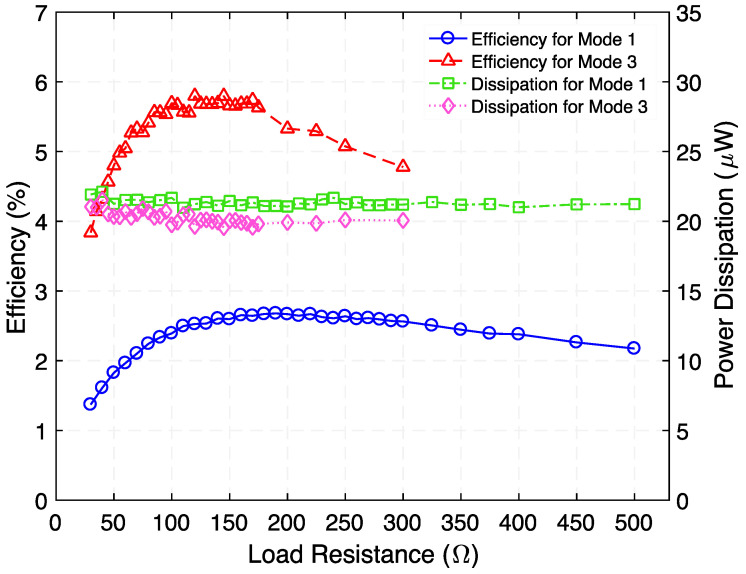
Measured efficiency and power dissipation as a function of load resistance when the step-down transformer is operated at 728 Hz (Mode 1) and 1002 Hz (Mode 3); the applied input power is 500 µW.

Following this, the rms voltage across and time-average power delivered to a variable load resistance were measured, as reported in Figure 13. The results indicate that the power is maximized at 0.581 µW at 230 Ω and at 1.213 µW at 95 Ω for Modes 1 and 3, respectively. Making use of this measured time-average power, the efficiency and power dissipation of the step-down transformer as a function of load resistance were estimated, as shown in Figure 14. As seen in Figure 14, efficiency peaks of 2.67% (Mode 1) and 5.79% (Mode 3) are reached when load resistances of 190 Ω and 120 Ω are used, respectively. The power dissipation associated with these peaks are 21.6 µW (Mode 1) and 20.7 µW (Mode 3).

**Figure 15:**
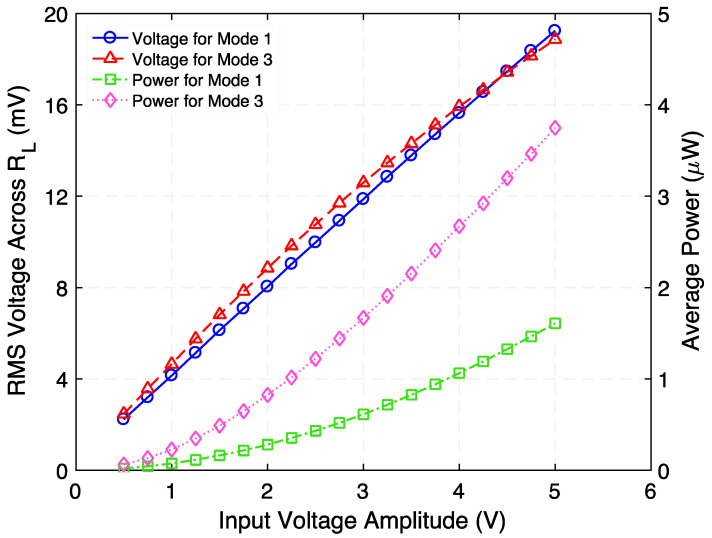
Measured rms voltage across and time-average power delivered to corresponding optimum load resistance as a function of input voltage when the step-down transformer is operated at 728 Hz (Mode 1) and 1002 Hz (Mode 3).

Figure 15 presents the rms voltage across and time-average power delivered to load resistance as a function of input voltage when the step-down transformer is operated at 728 Hz (Mode 1) and 1002 Hz (Mode 3). It is important to note that the optimum load resistance used was the one that maximizes the power transfer, specifically: 230 Ω and 95 Ω for Mode 1 and Mode 3, respectively. These results correlate satisfactorily with the quadratic behavior of the system’s output power.

**Figure 16:**
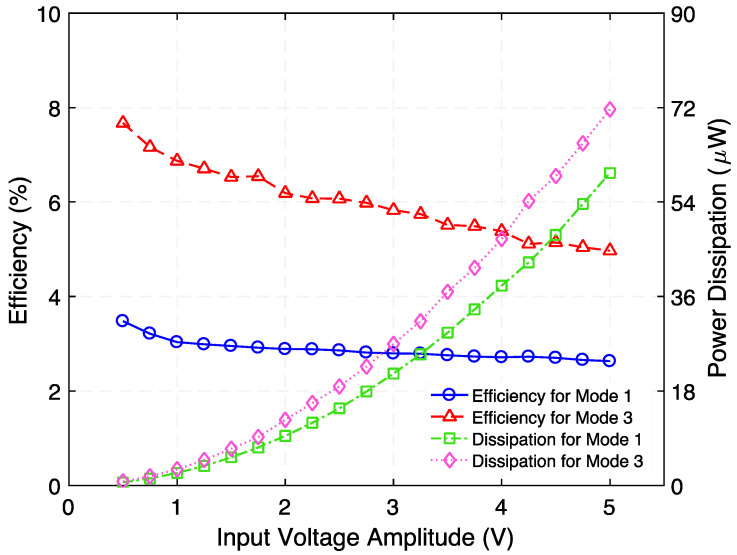
Measured efficiency and power dissipation as a function of input voltage of the step-down transformer when load resistances and resonance frequencies are 230 Ω, 95 Ω, and 728 Hz (Mode 1), 1002 Hz (Mode 3), respectively.

Furthermore, the efficiency and power dissipation as a function of the input voltage of the loaded (with corresponding optimum load resistance) step-down transformer are presented in Figure 16. Material and topology optimization for the mechanical resonator (including piezoelements) and magnetic coil, respectively, would be needed to improve efficiency.

**Figure 17:**
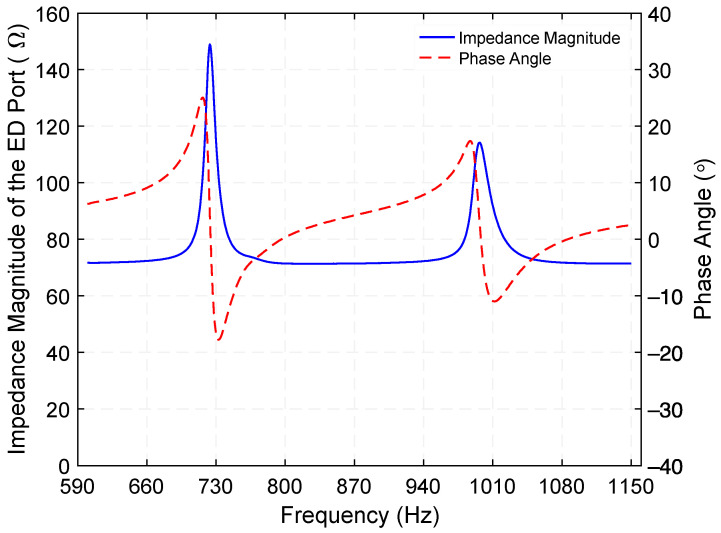
Measured electrical impedance magnitude and phase angle of the electrodynamical (ED) port; an excitation of 500 mV is applied while the piezoelectric (PE) port is open.

Finally, additional measurements of the electrical impedance of the ED port were performed using a precision impedance analyzer (Hewlett Packard 4294A). Figure 17 shows the electrical impedance magnitude and phase angle of the ED port when the PE port is open.

It can be seen from Figure 17 that the electrical response of the ED port is dominated by a first peak impedance of 148.3 Ω at 724.4 Hz and a second peak of 113.4 Ω at 998 Hz, for the resonance modes 1 and 3, respectively. Despite the fact that these peak impedances are slightly different from the optimum load resistances, a significant correlation between these values was found. As mentioned in the Prototype Fabrication subsection, the DC resistance of the electromagnetic coil (71 Ω) supports these results. Regarding the phase angle, it approaches 0 degrees at the resonance frequencies and, as expected in an inductive circuit, the current lags behind the voltage, resulting in a positive phase angle.

### 4.4. Charging Characteristics

**Figure 18:**
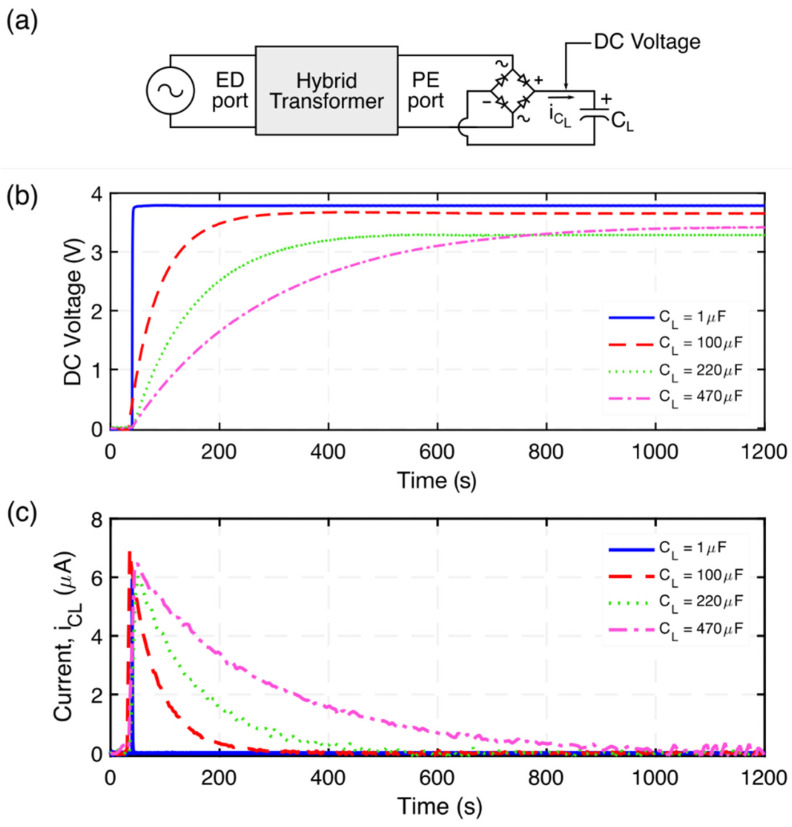
(**a**) Schematic diagram of the experimental setup used to explore the charging characteristics of the hybrid transformer, (**b**) measured DC voltage for various capacitors generated by the transformer operating under 500 mV_p-p_ at a 1015 Hz driving frequency, (**c**) capacitor current when charging; calculated using *i* = C dv/dt.

To explore the charging characteristics of the proposed hybrid transformer, a set of experiments were conducted using the step-up hybrid transformer along with 4 diodes and a capacitor, to rectifier and store the stepped-up AC voltage and DC output voltage, respectively. The system implementation is shown in Figure 18a. The demonstrated system is configured as an AC-DC step-up converter for low-voltage signals to charge a capacitor. The input signal is provided by an arbitrary waveform generator DAD board, generating 500 mV_p-p_ at a 1015 Hz (Mode 3) driving frequency. The power supply output is connected to the ED port of the hybrid electromechanical transformer. The stepped-up voltage (output voltage across the PE port of the transformer) is directly connected to a full wave rectifier bridge comprised by 4 diodes (1N4003). As a result, the rectified voltage will be stored at the capacitor. Four capacitor values were used to explore the charging characteristics of the transformer, namely, 1 µF, 100 µF, 220 µF, and 470 µF. Figure 18b presents the charging characteristics of the proposed hybrid transformer.

## 5. Conclusions

A hybrid electromechanical transformer able to perform reversible power conversion (step-up and step-down transformer type) is fabricated and characterized. This paper serves as a preliminary investigation of the feasibility of a hybrid electromechanical transformer concept as an alternative, low dissipation (0.9 µW), and low-profile (1.65 mm) to various single transduction transformers (e.g., piezoelectric, electromagnetic/electrodynamic), as well as to conventional magnetic transformer in certain applications. For applications, one may envision matching the frequency of the power source to the resonant frequency of the transformer. Alternatively, as is done in PTs, for the amplification of DC or quasi-DC power waveforms, one can use pulse/chopper circuits to operate the transformer at the resonance frequency. The hybrid electromechanical transformer described in this work demonstrated the feasibility of combining both electrodynamic transduction and piezoelectric transduction by means of mechanical resonator to transfer electrical energy from a circuit to another.

The device demonstrated an open-circuit voltage gain of 31.4 (29.9 dB) and 48.7 (33.7 dB) at 729.5 Hz and 1015 Hz resonance frequencies, respectively, and when operating as a step-up transformer type. In addition, power transfer efficiency peaks and associated dissipation of 9.6%, 1.7 µW (Mode 1) and 11.8%, 0.9 µW (Mode 3), respectively, are obtained when load resistances of 675 kΩ and 875 kΩ are connected to the output port of the step-up transformer. The effective electromechanical coupling coefficient of the hybrid transducer PE port reaches the maximum value of 12.2% and 16.4%, corresponding to its resonance modes 1 and 3, respectively.

Regarding the step-down transformer operation, the presented device achieved a 0.0097 (−40.2 dB) and 0.0128 (−37.8 dB) open-circuit voltage gain at 728 Hz (Mode 1) and 1002 Hz (Mode 3) resonance frequencies, respectively.

Future works include vacuum testing of the transformer, as well as a device optimization study to improve the power transfer efficiency and integration of this hybrid transformer with an ultra-low-consumption interface, such as wake-up radio or start-up converter. As stated above, the efficiency can be significatively improved by optimized dimensions of piezoelectric elements and better design of mechanical suspension and magnetic circuit to reduce power losses. Table 1 summarizes the compared performance, i.e., transducer type, voltage gain, driving frequency, and power density of the proposed hybrid electromechanical transformer with similar transducers reported in literature.

## 6. Patents

A provisional patent application entitled “Hybrid Electromechanical Transformer” and filed in the United States Patent and Trademark Office (USPTO) under Serial No. 63/223,756, results from the work reported in this manuscript.

## Figures and Tables

**Table 1:** Summary of performance compared to similar transducers.

Parameter	[15]	[16]	[20]	This Work
Transducer type	PE	PE	ED	Hybrid
Voltage gain	58 mV/V	23.2 V/V	12 V/V	48.7 V/V
Driving frequency	36.3 kHz	55.8 kHz	22 Hz	1015 Hz
Power density	N/A^*^	1.27 µW/cm^3 a^	1.87 µW/cm^3 a^	1.89 µW/cm^3^

* Not reported in reference, ^a^ Calculated from reference, PE = Piezoelectric, ED = Electrodynamic.
